# Digital Anorectal Examination to Self-detect Primary Syphilis: A Prospective Cohort Study

**DOI:** 10.1093/infdis/jiaf628

**Published:** 2025-12-11

**Authors:** Julien Tran, Kate Maddaford, Jason J Ong, Ei T Aung, Christopher K Fairley, Eric P F Chow

**Affiliations:** Melbourne Sexual Health Centre, Alfred Health, Melbourne, Victoria, Australia; School of Translational Medicine, Faculty of Medicine, Nursing and Health Sciences, Monash University, Melbourne, Victoria, Australia; Melbourne Sexual Health Centre, Alfred Health, Melbourne, Victoria, Australia; School of Translational Medicine, Faculty of Medicine, Nursing and Health Sciences, Monash University, Melbourne, Victoria, Australia; Melbourne Sexual Health Centre, Alfred Health, Melbourne, Victoria, Australia; School of Translational Medicine, Faculty of Medicine, Nursing and Health Sciences, Monash University, Melbourne, Victoria, Australia; Melbourne Sexual Health Centre, Alfred Health, Melbourne, Victoria, Australia; School of Translational Medicine, Faculty of Medicine, Nursing and Health Sciences, Monash University, Melbourne, Victoria, Australia; Melbourne Sexual Health Centre, Alfred Health, Melbourne, Victoria, Australia; School of Translational Medicine, Faculty of Medicine, Nursing and Health Sciences, Monash University, Melbourne, Victoria, Australia; Melbourne Sexual Health Centre, Alfred Health, Melbourne, Victoria, Australia; School of Translational Medicine, Faculty of Medicine, Nursing and Health Sciences, Monash University, Melbourne, Victoria, Australia; Centre for Epidemiology and Biostatistics, Melbourne School of Population and Global Health, The University of Melbourne, Melbourne, Victoria, Australia

**Keywords:** prevention, DARE, digital anorectal examination, syphilis, sexually transmissible infections

## Abstract

**Background:**

Primary anorectal syphilis may go unnoticed in men who have sex with men (MSM) engaging in receptive anal sex. This study examined whether weekly digital anorectal examination (DARE) could help men self-detect abnormalities indicative of primary anorectal syphilis.

**Methods:**

A cohort study of MSM aged ≥18 years who engage in receptive anal sex was conducted at the Melbourne Sexual Health Centre from 9 March 2022 to 4 August 2023. Participants received instructions on how to perform DARE, along with weekly text reminders for 48 weeks. Those who self-detected abnormalities were advised to seek clinical consultation. The primary outcome was the proportion of syphilis cases detected via DARE. Secondary outcomes included reports of DARE-related abnormalities, adherence, and experiences.

**Results:**

Of the 222 men recruited, six men (2.7%; 95% CI: 1.0–5.8) were diagnosed with syphilis—1 primary anorectal infection detected by DARE, 2 secondary infections, and 3 early latent syphilis infections. There were 32 clinical consultations prompted by DARE. On average, men performed 78.2% (95% CI: 77.3–79.0) of their weekly DARE which showed no significant variation over time (*P*_trend_ = 0.26). Most found DARE easy to perform (>95.0%) and would continue performing it if recommended for early syphilis detection (77.6%).

**Conclusions:**

Men's high adherence to performing we DARE suggests that it may complement routine screening for primary anorectal syphilis. However, its sensitivity may be limited, as 5 of 6 early syphilis cases did not have primary lesions that were self-detected by the 5 men.

## Teaser

This cohort of 222 MSM showed high adherence and acceptability for weekly digital anorectal examination, and it may complement routine syphilis screening, although its sensitivity is limited.

Syphilis disproportionately affects gay, bisexual, and other men who have sex with men (MSM) [1–3]. Despite regular testing, treatment, and tracing of contacts [4–6], syphilis infections continue to rise in this population. Cases may further increase, given the recent rise in condomless anal sex in MSM, which has coincided with the availability of human immunodeficiency virus (HIV) preexposure prophylaxis (PrEP) and awareness of treatment as prevention [7–9].

Approximately 40% of individuals with a primary syphilis infection present with painless lesions (chancres) that can, on average, range from 0.3 to 3.0 cm in size at the anatomical site of infection about 3 weeks after sexual exposure [10, 11]. If untreated, these lesions resolve in 3–6 weeks [12]. However, weeks to months later, secondary syphilis may develop and is characterized by symptoms such as fever, headache, and maculopapular rashes [10]. If individuals are untreated during the first 2 years of infection, they are classified as having early latent syphilis, a stage during which there is a risk of neurosyphilis. Therefore, early detection is important in preventing serious issues such as neurosyphilis and in reducing the duration of infectiousness and risk of onward transmission.

Efforts to control syphilis through regular testing for sexually transmissible infections (STIs) have reduced the duration of infectiousness [13], but the incidence of syphilis has not reduced, indicating that additional steps are required. Recent research has highlighted that some anorectal cases of primary syphilis may be being missed. In this research, MSM who reported receptive anal sex in the past 3 months had 3.9 times higher odds of presenting with secondary rather than primary syphilis [14]. This suggests that some men engaging in receptive anal sex might not notice their anorectal lesions, allowing the infection to progress from the primary to the secondary stage.

Some researchers have suggested that performing digital anorectal examination (DARE) could identify anorectal lesions [15] and result in earlier presentation to healthcare [16]. These examinations involve individuals using their finger(s) to feel in and around their anus and visually checking their perianal area (5 cm diameter from the anal verge) for abnormalities [17]. This method has been promoted for the early diagnosis of anal cancer but not specifically for STIs [18]. While performing weekly DARE is highly acceptable [17, 19–22] and well adhered to over a period of 12 weeks among MSM [17], there are no data on whether weekly DARE can self-detect abnormalities related to primary syphilis or how often unrelated abnormalities are found. Therefore, we conducted a 48-week prospective cohort study of MSM to examine whether men who perform DARE weekly could self-detect abnormalities for the early detection of primary anorectal syphilis.

## METHODS

### Study Setting and Recruitment

Between 09 March 2022 and 05 July 2024, we conducted a 48-week prospective cohort study to examine whether weekly DARE could detect primary anorectal syphilis among MSM attending the Melbourne Sexual Health Centre (MSHC) in Australia. The Alfred Hospital Ethics Committee approved this study (646/21). Men were eligible if they were 18 years or older, had receptive anal sex in the past 12 months, and had sufficient English to provide informed consent. Men were ineligible if they planned to travel internationally for more than 3 months during the study or were enrolled in another study on syphilis prevention. Clinicians referred eligible participants to the research team who explained the study's procedures and requirements before enrollment. The research team met with participants on the same day, arranged a convenient appointment time, or recruited them over the phone. Written or verbal informed consent to participate was provided by all participants. Participants underwent routine STI testing—including gonorrhea, chlamydia, syphilis, and HIV—at enrollment (ie, baseline). They were treated in accordance with the Australian STI management guidelines before study commencement [23].

During enrollment, participants were asked to watch an animated instructional video on how to perform DARE. To support weekly DAREs, we adapted existing instructions into a visually focused sheet and produced a matching video tutorial narrated by a nurse. Participants were provided with the video link and the illustrated sheet for future reference. They were then shown images of a range of syphilis lesions, rashes, and other signs (eg, anal condylomata) available on the MSHC website. Participants were also given a mini hand-held mirror to check for abnormalities and advised to contact the research team if they identified any. Participants were asked to perform weekly DARE using the instructional video or sheet for 48 weeks (Supplementary Figure 1).

### Baseline

At baseline, participants completed a self-administered online survey about their previous experiences with DARE, sexual practices in the past 3 months, and previous history of syphilis infection. They were asked to attend regular 3-monthly STI testing at MSHC or with their general practitioner (GP); however, they could also test anytime during the study if they had symptoms.

### Follow-Up

We sent automated weekly text messages to all participants asking if they performed DARE during that week, to which they could reply: “Yes” or “No.” If they did not reply, this was recorded as a “No response.” If participants did not respond to the weekly text reminders for 4 consecutive weeks, they were prompted with follow-up phone calls and/or emails from the research team. If they still did not respond to the reminders for 2 more consecutive weeks after the prompting, they were defined as lost to follow-up (LTFU). Throughout the 48-week period, participants were sent quarterly surveys via text message or email if they were overseas. The surveys collected data on their sexual practices (number of partners and condom use) and experiences with performing DARE, including action taken after finding abnormalities, for example, by consulting a GP. At Week 48 (end of study), they were asked additional questions about how and where they performed DARE, reasons why they did not perform DARE, whether they performed DARE on their partner(s), and their preferences and suggestions regarding DARE.

Participants were considered as having completed the study if they actively participated from the beginning to the end of the designated study period (ie, Week 48), had not withdrawn from the study or were not LTFU, and provided essential data from the required tests and surveys. Participants received an AU$50 voucher at Weeks 24 and 48.

If participants had STI testing at their GPs, they were asked to provide the research team with these test results. If this was not possible, a research team member requested these test results from their GPs, using the signed permission provided during study enrollment.

### Clinical Consultations for Anorectal Abnormalities

Participants who identified abnormalities during DARE were instructed to contact the research team to schedule a review appointment with a clinician. The review included an examination, anoscopy, serological testing, and/or clinician-collected anal swabs, if intraanal abnormalities were present. Testing for other STIs was conducted if deemed necessary by the attending clinician. Participants who underwent review at their GP informed the research team by responding to quarterly surveys. By auditing these surveys and participants’ medical records, a research team member (J. T./K. M.) contacted those who presented to their GP or MSHC with anorectal abnormalities but had not contacted the research team to clarify whether the abnormalities were detected during DARE.

### Testing and Diagnosis of Syphilis

We defined infectious syphilis as <2 years as per the definition from the Australian Department of Health and Aged Care [24]. Syphilis was staged as primary or secondary through findings from clinical examinations and confirmed with a positive laboratory result for *Treponema pallidum* polymerase chain reaction (PCR) from a syphilitic lesion and/or from serological testing. Early latent syphilis was defined as seroconversion within the past 2 years. A reinfection of syphilis was defined as at least a 4-fold increase in rapid plasma reagin (RPR). In our study, *T. pallidum* PCR was performed at the Victorian Infectious Diseases Reference Laboratory, using a TaqMan real-time PCR assay targeting the *polA* gene of *T. pallidum.* Serological testing for syphilis involved chemiluminescence immunoassay (DiaSorin LIAISON), *T. pallidum* particle agglutination (Fujirebio), and RPR (Becton Dickinson).

### Outcomes

The primary outcomes were the number and proportion of men who detected primary syphilis through DARE. Secondary outcomes include the number and proportion of men who found any abnormalities while performing DARE or had a clinical consultation after finding any abnormality while performing DARE, adherence to performing weekly DARE, and their experiences and suggestions regarding DARE.

### Analysis

To obtain the appropriate sample size to show that primary anorectal syphilis could be detected by DARE, we estimated an incidence of 5% per year [2], equivalent to approximately 10 cases per 100 person-years in a cohort of 200 MSM followed for 1 year. We expected that about 4 in 10 of these cases might be identified through DARE. We calculated the median and interquartile range (IQR) for continuous variables and proportions and 95% CI using binomial exact methods. Syphilis incidence was calculated as the number of new syphilis cases divided by the number of person-years-at-risk. Person-years-at-risk was defined as the time between the first and last test of syphilis during the study and expressed as 100 person-years [25]. This estimate of incidence included the first and last test of syphilis for those who withdrew or were LTFU. We calculated adherence to weekly DARE by dividing the number of weeks DARE was performed by the total number of weeks in the study's observation period [17]. For participants who withdrew from the study or were LTFU, adherence was calculated only up to the date of their withdrawal or LTFU. We used linear regression analysis to examine temporal trends at 12-week intervals in the average number of times weekly DARE was performed, and number of partners for anal sex (with and without condoms). Temporal trends in whether men found DARE easy and comfortable to perform and whether they detected any abnormalities during DARE were examined using chi-square trend analysis. Statistical significance was defined as *P* < .05. All analyses were conducted using Stata (Version 17).

## RESULTS

**Figure 1.**
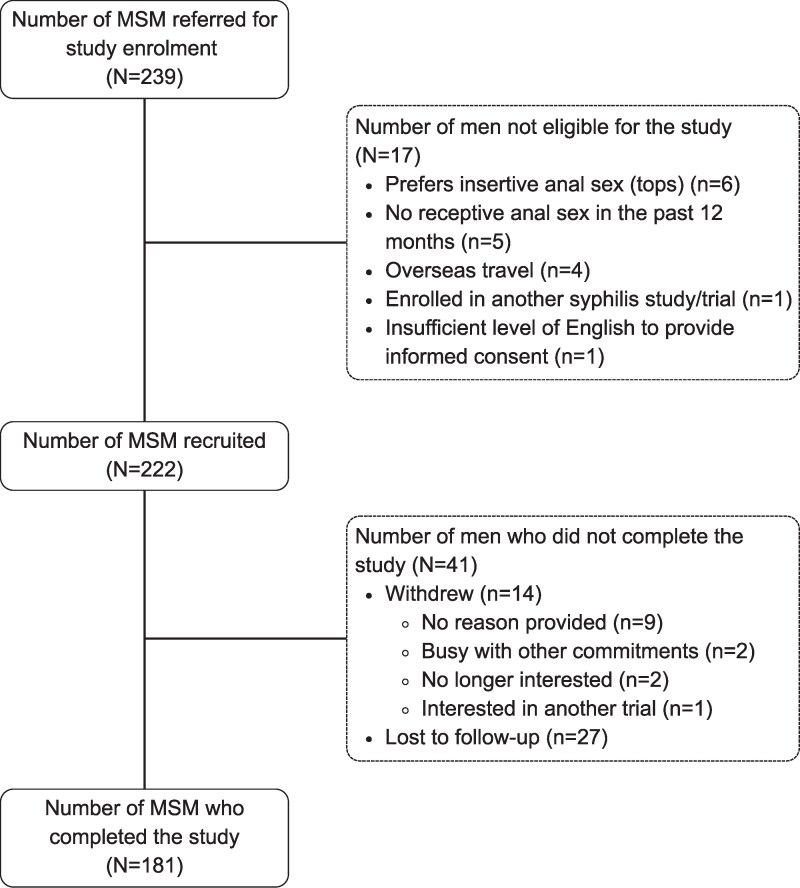
Flowchart illustrating the recruitment process for the cohort study. MSM, men who have sex with men.

Between 9 March 2022 and 4 August 2023, a total of 239 men were referred to the research team (Figure 1). Of these 239 men, 17 (7.1%) were ineligible; reasons for study ineligibility are provided in Figure 1.

A total of 222 men met our inclusion criteria and were enrolled in the study. The median age was 31 years (IQR = 27–38 years) (Table 1). Most were users with PrEP (61.3%, 136/222), followed by users with HIV-negative non-PrEP (22.5%, 50/222), and people living with HIV (16.2%, 36/222). Most were born in Oceania (41.0%, 91/222), followed by Asia (32.0%, 71/222). About a third (33.8%, 75/222) had a previous history of syphilis infection. At enrollment, 1 man had secondary syphilis, and 2 had early latent syphilis and they were treated before study commencement.

**Table 1.** Baseline Characteristics of 222 MSM Who Have Had Receptive Anal Sex in the Past 12 mo

Characteristics	Statistics
Age, median (IQR)	31 (27–38)
Region of birth, n (%)	
Oceania	91 (41.0%)
Asia	71 (32.0%)
Europe	25 (11.3%)
Middle East or Africa	5 (2.2%)
North America	2 (0.9%)
South America or the Caribbean	28 (12.6%)
HIV status or PrEP use, n (%)	
HIV-negative and not using PrEP	50 (22.5%)
HIV-negative and using PrEP	136 (61.3%)
People living with HIV	36 (16.2%)
Previous history of syphilis infection, n (%)	
Yes	75 (33.8%)
No	147 (66.2%)
Role in anal sex, n (%)	
Versatile^a^	167(75.2%)
Receptive only	55 (24.8%)
Currently performing digital anorectal self-examination regularly, n (%)	
Yes	9 (4.1%)
No	213 (95.9%)
Ever performed a digital anorectal self-examination, n (%)	
Yes	70 (31.5%)
No	152 (68.5%)
Number of male partners with whom they engaged in receptive anal sex in the past 3 mo	
With a condom, median (IQR)	2 (1–3)
Without a condom, median (IQR)	3 (1–6)
Did not have receptive anal sex, n (%)	15 (6.8%)

Abbreviations: IQR, interquartile range; HIV, human immunodeficiency virus; PrEP, preexposure prophylaxis.

^a^Men who identified as versatile in their roles in anal sex engaged in both receptive and insertive anal sex.

At baseline, men reported a median of 3 (IQR = 1–6) condomless receptive anal sex partners and 2 (IQR = 1–3) receptive anal sex partners with condoms in the past 3 months (Table 1). About 31.5% (70/222) of men reported they had ever performed DARE, and 4.1% (9/222) were currently performing DARE regularly.

Of the 222 recruited men, 27 men were LTFU, and 14 men withdrew from our study. Of the 14 men who withdrew, 9 did not provide a reason, 2 were busy with other commitments, 2 were no longer interested, and 1 changed to another study at the MSHC. A total of 181 men completed the study.

Six cases of syphilis were diagnosed in 6 men including 1 primary (anorectal), 2 secondary, and 3 early latent syphilis infections (Table 2). Four cases of syphilis were diagnosed at MSHC, and 2 were diagnosed by a GP. The overall incidence of syphilis was 5.0 (95% CI: 2.2–11.1) per 100-person-years. Of the 6 syphilis cases, 1 case (16.7%, 95% CI: .4%–64.1%) was identified by DARE, 3 cases were diagnosed through screening, and 2 cases presented with secondary syphilis. The syphilis case identified by DARE was primary anorectal syphilis, and the participant was diagnosed after he found some perianal and intra-anal abnormalities while performing DARE and subsequently attended MSHC for a review. The participant thought his abnormalities were hemorrhoids, but they were confirmed as syphilitic lesions by a clinician. This man had a positive PCR for *T. pallidum* from an anorectal swab and was also positive for syphilis through serological testing, with RPR titer of 1:1, showing detectable antibodies at serum dilution of 1:1. He had performed 91% (41/45 weeks) of the weekly DARE from the date of his recruitment up until the date of attendance at MSHC, including a DARE within a week of the DARE that led to his diagnosis.

**Table 2.** Details of Testing and Diagnosis of Syphilis for Six Participants and Their Adherence to Weekly DARE

Participant^a^	Diagnosis	Reinfection	Time Between Recruitment And Diagnosis, And Location And Reason For Consultation	Syphilis Serology	Syphilis PCR	RPR On Days of Testing	RPR At Previous Consultation	Adherence To DARE From Enrollment to Initial Visit, n/N %	Number of Days Between Last DARE And Day of Diagnosis	Symptoms	Total Syphilis Tests During Study, Excluding Baseline
A	Early latent syphilis	Yes	244 d GP Asymptomatic testing	Positive	Not done	1:16	Nonreactive	50% 17/34 wk	14 d	No (Day 1)	4
B	Secondary syphilis	No	220 d MSHC Asymptomatic testing	Positive	Not done	1:64	Nonreactive	84% adherence 26/31 wk	21 d	No (Day 1) Yes (Day 8 recall) Symptoms: Maculopapular rash on torso	4
C	Primary anorectal syphilis	No	316 d Returned to MSHC because the participant identified a lump when performing a DARE	Positive	Positive	1:1	Nonreactive	91% 41/45 wk	7 d	Yes (Day 1) Symptoms: anorectal pain, bleeding, and itch past 7 d	2
D	Early latent syphilis	Yes	201 d MSHC Contact of infection (gonorrhea and chlamydia)	Positive	Not done	1:32	1:8	71% 20/28 wk	14 d	No (Day 1) No (Day 29 recall) No rash or lesions	2
E	Secondary syphilis	Yes	240 d MSHC Asymptomatic testing	Positive	Not done	1:64	1:4	100% 34/34 wk	7 d	No (Day 1) Yes (Day 6 recall) Symptoms: secondary syphilitic rash on hands and arms	2
F	Early latent syphilis	Yes	129 d (tested at GP) 142 d (referred to MSHC for treatment)	Positive	Not done	1:1024	1:4	100% 18/18 wk	7 d	No (Day 1) No (Day 14 recall)	2

Proportion for DARE adherence is calculated using “yes’ responses to weekly text message reminders from the date of enrollment and date of consultation. Day 1 represents the date of the consultation to maintain confidentiality and avoid disclosing potentially identifying information about the participants. The term “Day *n* recall” indicates the number of days elapsed since Day 1.

Abbreviations: DARE, digital anorectal examination; GP, general practitioner; MSHC, Melbourne Sexual Health Centre; RPR, rapid plasma reagin; PCR, polymerase chain reaction; n, number of weeks DARE performed, N, number of weeks DARE could potentially have been performed.

^a^Participants’ study identification numbers were altered to avoid providing any identifiable information.

**Figure 2.**
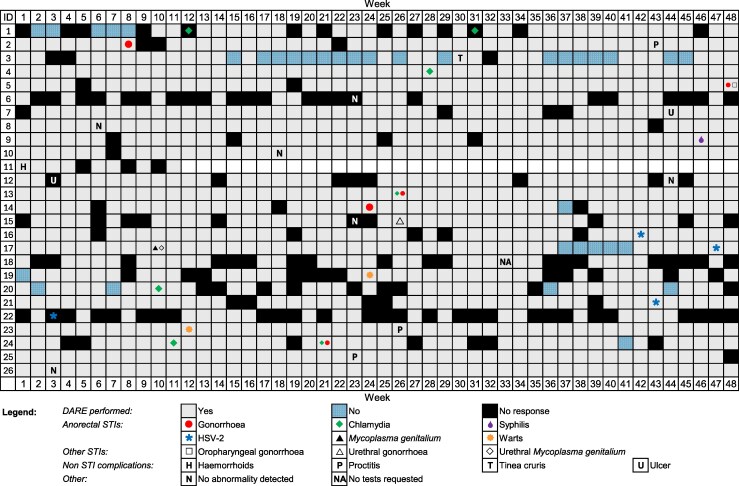
Self-reported adherence to performing digital anorectal examination (DARE) over 48 wk, and diagnoses of sexually transmissible infections and non-STI complications for 26 men and the week they attended a clinical consultation with abnormalities detected during DARE. Note: Participants’ study identification numbers were altered to avoid providing any identifiable information. Six of 31 consultations with STI testing did not result in a confirmed diagnosis of syphilis or other anal STIs, resulting in a false positive rate of approximately 19%. STI, sexually transmissible infection.

There were a total of 32 clinical consultations from 25 men without syphilis who attended clinical consultations with abnormalities they found when they performed DARE. Of the 32 consultations, 28 were at MSHC (87.5%) and 4 were at a GP (12.5%). Of the 32 consultations, 19 anorectal STIs were diagnosed among 15 men (Figure 2). This included 7 cases of chlamydia (21.9%, 95% CI: 9.2%–40.0%), 5 gonorrhea (15.6%, 95% CI: 5.3%–32.8%), 4 herpes (12.5%, 95% CI: 3.5%–30.0%), 2 warts (6.3%, 95% CI: .7%–20.8%), and 1 *Mycoplasma genitalium* (3.1%, 95% CI: .1%–16.2%). Other conditions included 2 pathogen-negative ulcers (6.3%, 95% CI: .7%–20.8%), 3 pathogen-negative proctitis (9.4%, 95% CI: 1.9%–25.0%), and 1 hemorrhoid (3.1%, 95% CI: .1%–16.2%).

DARE was performed by 222 men in 7372 weeks out of 9433 weeks of observation, amounting to 78.2% (95% CI: 77.3%–79.0%) adherence to weekly DARE. Of the men, 58.1% (129/222) had 75%–100% adherence to weekly DARE, 15.8% (35/222) had 50%–74% adherence, 9.9% (22/222) had 26%–49% adherence, and 16.2% (36/222) had 0%–24% adherence. On average, men performed DARE 9.3 times every 12 weeks, and this average did not change over the 48-week period (*P*_trend_ = 0.261). The majority of the men found performing DARE to be easy (>95%) and comfortable (>85%) and this did not significantly change over the study period (Table 3). The average number of days between finding an abnormality and the clinical consultation was 3.4 (SD = 3.2) days. The proportion of men who found abnormalities, such as bleeding, discharge and lumps while performing DARE increased over time in the study (*P*_trend_ = 0.017). A majority of men who performed a DARE and reported an abnormality sought help (88.1%, 59/67), and this did not significantly change throughout the study (*P*_trend_ = 0.175).

**Table 3.** Experiences With DARE and Sexual Practices Throughout the Study Period

Details	Week 12 (N = 128)	Week 24 (N = 132)	Week 36 (N = 95)	Week 48 (N = 125)	
	n (%)	n (%)	n (%)	n (%)	*P* value
Experiences with DARE in the past 3 mo					
Men found performing DARE to be					
Easy	124 (96.9)	126 (95.4)	90 (96.8)	119 (95.2)	.616*
Difficult	4 (3.1)	6 (4.6)	3 (3.2)	6 (4.8)	
Men found performing DARE to be					
Comfortable	112 (87.5)	114 (86.4)	82 (86.3)	111 (88.8)	.774*
Uncomfortable	16 (12.5)	18 (13.6)	13 (13.7)	14 (11.2)	
Finding abnormalities through DARE					.017*
Men who did not find abnormalities when they performed a DARE	116 (90.6)	117 (88.6)	78 (82.1)	102 (81.6)	
Men who found abnormalities when they performed a DARE	12 (9.4)	15 (11.4)	17 (17.9)	23 (18.4)	
Abnormalities found**^a^**					
Bleeding	2 (16.7)	1(6.6)	2 (11.8)	3 (13.0)	
Discharge	3 (25.0)	1(6.6)	1 (5.9)	4 (17.4)	
Lump(s)	8 (66.7)	7 (46.7)	7 (41.2)	14 (60.9)	
Pain	4 (33.4)	5 (33.3)	6 (35.3)	4 (17.4)	
Ulcer(s)	1 (8.3)	1 (6.6)	2 (11.8)	3 (13.0)	
Other (eg, rash, skin tag, hemorrhoid)	1 (8.3)	3 (20.0)	1 (5.9)	1 (4.4)	
Reactions after finding abnormalities from performing a DARE**^a^**					
Angry	0 (0.0)	1 (6.6)	0 (0.0)	1 (4.3)	
Anxious	6 (50.0)	5 (33.3)	9 (52.9)	10 (43.5)	
Scared	0 (0.0)	3 (20.0)	1 (5.9)	3 (13.0)	
Worried	6 (50.0)	8 (53.3)	9 (52.9)	13 (56.5)	
Neutral	5 (41.7)	5 (33.3)	7 (41.2)	6 (26.0)	
Men who sought help after finding abnormalities from performing DARE	9/12 (75.0)	14/15 (93.3)	14/17 (82.4)	22/23 (95.7)	.175*
Actions after finding abnormalities from performing a DARE^a^					
Consulted a doctor or a nurse at MSHC	3 (25.0)	5 (33.3)	3 (17.6)	9 (39.1)	
Consulted a GP	3 (25.0)	7 (46.7)	7 (41.2)	8 (34.8)	
Contacted the research study team	3 (25.0)	5 (33.3)	3 (17.6)	7 (30.4)	
Did an online search for more information	4 (33.4)	7 (46.7	5 (29.4)	5 (21.7)	
Talked to friend(s)	2 (16.7)	3 (20.0)	1 (5.9)	3 (13.0)	
Talked to sexual partner(s)	1 (8.3)	3 (20.0)	4 (23.5)	6 (26.0)	
Number of days until a DARE-related clinical consultation after finding abnormalities, mean (95% CI)	5.3 (2.9–7.6)	2.9 (1.2–4.6)	4.7 (2.9–6.6)	2.4 (1.0–3.9)	
Number of days until a DARE-related clinical consultation after finding abnormalities, median (IQR)	3 (2–7)	2 (1–4)	4 (1–7)	2 (1–3)	
Sexual practices in the past 3 mo					
Number of sexual partners for receptive anal sex					
With condoms, mean (95% CI)	3.0 (2.0–4.0)	2.3 (1.3–3.2)	2.6 (1.6–3.7)	2.3 (1.3–3.3)	.474*
With condoms, median (IQR)	2 (1–3)	2 (1–3)	2 (1–3)	2 (1–3)	
Without condoms, mean (95% CI)	5.3 (3.8–7.0)	6.4 (4.9–8.0)	6.1 (4.2–8.0)	6.0 (4.3–7.6)	.692*
Without condoms, median (IQR)	3 (2–7)	3 (1–8)	3 (2–7)	4 (1–6)	

Abbreviations: DARE, digital anorectal examination; IQR, interquartile range; OR, odds ratio; GP, general practitioner.

^a^Multiple answers were permitted, and proportions may exceed 100%.

^*^ *P* for temporal trend from Week 12 through to Week 48.

Of the 181 men who completed the study, 125 (69.0%) completed the Week 48 survey. Among these 125 men, squatting was the most common position used when they performed a DARE (44.8%; 95% CI: 35.9%–54.0%, n = 56) (Supplementary Table 1). When the men did not perform a DARE, common reasons included forgetting about it (32.0%, 40/125) and being busy with work and other commitments (24.8%, 31/125). Furthermore, 35.2% (44/125) performed DARE on their partner, although this practice was not recommended as part of the study, and 9.1% (4/44) found abnormalities on their partner. Most men would continue performing DARE if it were recommended for detecting syphilis (77.6%, 97/125) and for reasons related to their health, such as detecting conditions early, seeking treatment, and maintaining sexual health (88.9%, 80/90). Options to increase DARE among men and other suggestions by participants are presented in Supplementary Table 2.

## DISCUSSION

To our knowledge, this is the first study to examine the effectiveness of DARE in detecting primary anorectal syphilis among MSM. Of the 222 men in the study, one case of primary anorectal syphilis was identified by DARE in addition to routine syphilis screening. While we demonstrated that DARE could detect primary anorectal syphilis, it appears to have missed most early syphilis infections. Men adhered well to weekly self-DARE for the entire study, suggesting it was acceptable to most men. Encouragingly, the majority of participants sought health care and syphilis testing after identifying an abnormality through DARE.

Our data suggests that even with weekly DARE, some primary infections were presumably missed, given that the 2 cases of secondary syphilis and 3 cases of early latent syphilis with relatively high RPRs were not detected by DARE. This finding implies that since cases did not report penile chancres, the men may have had anorectal lesions that could not be detected by DARE or that these men did not have primary anorectal lesions. Of the 6 cases of syphilis in our study, 4 had a previous history of infection, which can affect the likelihood of chancre development. For instance, a study conducted in 1953 in a New York prison where prisoners were inoculated with live *T. pallidum* found all individuals without a history of syphilis developed chancres at the site of inoculation [26]. In contrast, those with a previous history of syphilis had a lesser chance of developing chancres. In men with a previous history of syphilis, treatment was delayed for about 4 months following inoculation [26]. Interestingly, 4 of the 6 cases of syphilis in our study had syphilis previously. Future studies should, therefore, explore this issue in greater depth perhaps through comparing DARE in men with and without a history of syphilis who have a high number of sexual partners. Understanding the proportion of syphilis cases that DARE can detect could be crucial in determining its effectiveness as a population-level intervention.

It is possible that not performing DARE in the weeks before a syphilis diagnosis might have been responsible for men missing primary syphilis chancres. In the 5 syphilis cases that did not have primary lesions detected, 2 of the men were consistent with their weekly DARE, but 3 had not responded to text messages in the 2–3 weeks before their diagnosis. This suggests that the primary anorectal lesions may have been missed because DARE was performed too infrequently in these 3 men. The size of primary lesions can range from 0.5 to 3 cm [27 ], and these lesions can occur around 7–9 cm inward from the anal verge [28, 29]. Given that the average length of an adult male index finger is around 6.7 cm, measured from the base to the tip of the finger and about 4.4 cm from the tip to the second knuckle [30], it was possible that, even with full finger insertion, lesions were located beyond the reach of a finger during the examination. In our study, we instructed men to insert their finger up to the second knuckle rather than as far as possible, which may have led to lesions being missed because they were out of reach. It is also possible that men were unable to sufficiently rotate their finger to detect lesions.

Adherence to DARE among sexually active MSM is important for the self-detection of primary syphilis, given that primary syphilis lesions typically last 3–6 weeks [12, 31]. To date, only one other study by Aung et al. [17] has examined adherence to performing weekly DARE over a 12-week period among 30 MSM. Consistent with Aung et al.'s study [17], men in our study demonstrated relatively high adherence to DARE, performing, on average, 3 times every 4 weeks, despite some of the men feeling uncomfortable with performing DARE. However, unlike our study, theirs had approximately 20 times fewer observation time and did not detect any cases of syphilis. Our study showed that DARE can prompt early healthcare-seeking for syphilis diagnosis and treatment, but only a minority of cases were detected. While the 95% CI for detection included an upper bound of 64%, this should be interpreted with caution given the small number of syphilis cases.

Consistent weekly DARE may facilitate increased detection of anorectal STIs, particularly those that are asymptomatic and visually identifiable, such as painless ulcers or warts. While the study setting allowed for prompt follow-up, it remains uncertain whether such abnormalities would be detected more quickly in real-world contexts. Nonetheless, regular DARE practice may enhance men's familiarity with their own anatomy, potentially leading to earlier recognition of changes and timely healthcare seeking.

Our study has several limitations. First, about 20% of participants were either LTFU or had withdrawn from the study. These men may have lost interest in the study or found DARE unacceptable as an intervention. As the study population was drawn from a sexual health clinic, findings may not be fully generalizable to the broader MSM community. It is unclear how acceptable or feasible weekly DARE would be among MSM who do not routinely attend sexual health clinics, as they may differ in health-seeking behavior and risk perception. Second, recall bias may have occurred due to self-reported adherence to DARE, although we minimized this bias by prospectively collecting weekly data. Additionally, only 69% of participants provided feedback on their experiences with DARE, which introduces potential response bias. The perspectives of nonrespondents regarding their DARE-related experiences and suggestions may differ from those who responded. Fourth, the study began during the COVID-19 pandemic and continued through associated lockdown restrictions, which may have influenced sexual practices and STI risks [32, 33]. However, the syphilis incidence estimated in our cohort was similar to that of another Australian study [2].

## CONCLUSIONS

Our study provides proof of concept that DARE can detect syphilitic lesions, although most cases were still missed. DARE may serve as a simple and low-cost tool for early identification of other anorectal conditions. The high adherence suggests that men may be willing to incorporate DARE as part of their routine self-care. Further longitudinal research is warranted to evaluate its effectiveness for detecting primary syphilis in diverse settings and populations.

## Supplementary Material

jiaf628_Supplementary_Data

## Supplementary Data

Supplementary materials are available at *The Journal of Infectious Diseases* online (http://jid.oxfordjournals.org/). Supplementary materials consist of data provided by the author that are published to benefit the reader. The posted materials are not copyedited. The contents of all supplementary data are the sole responsibility of the authors. Questions or messages regarding errors should be addressed to the author.

## References

[1] Newman L, Rowley J, Vander Hoorn S, et al Global estimates of the prevalence and incidence of four curable sexually transmitted infections in 2012 based on systematic review and global reporting. PLoS One 2015; 10:e0143304. 26646541 10.1371/journal.pone.0143304 PMC4672879

[2] Aung ET, Fairley CK, Ong JJ, et al Incidence and risk factors for early syphilis among men who have sex with men in Australia, 2013–2019: a retrospective cohort study. Open Forum Infect Dis 2023; 10:ofad017. 36751651 10.1093/ofid/ofad017 PMC9898878

[3] Aung ET, Chow EPF. Testing and capturing difficult-to-access populations for syphilis control in Australia. Microbiology Australia 2024; 45:142–6.

[4] Zou H, Fairley CK, Guy R, Chen MY. The efficacy of clinic-based interventions aimed at increasing screening for bacterial sexually transmitted infections among men who have sex with men: a systematic review. Sex Transm Dis 2012; 39:382–7. 22504605 10.1097/OLQ.0b013e318248e3ff

[5] Bissessor M, Fairley CK, Leslie D, Howley K, Chen MY. Frequent screening for syphilis as part of HIV monitoring increases the detection of early asymptomatic syphilis among HIV-positive homosexual men. J Acquir Immune Defic Syndr 2010; 55:211–6. 20585261 10.1097/QAI.0b013e3181e583bf

[6] Chow EPF, Callander D, Fairley CK, et al Increased syphilis testing of men who have sex with men: greater detection of asymptomatic early syphilis and relative reduction in secondary syphilis. Clin Infect Dis 2017; 65:389–95. 28419198 10.1093/cid/cix326

[7] Holt M, MacGibbon J, Bear B, et al Trends in belief that HIV treatment prevents transmission among gay and bisexual men in Australia: results of national online surveys 2013–2019. AIDS Educ Prev 2021; 33:62–72. 33617321 10.1521/aeap.2021.33.1.62

[8] Holt M, Lea T, Mao L, et al Community-level changes in condom use and uptake of HIV pre-exposure prophylaxis by gay and bisexual men in Melbourne and Sydney, Australia: results of repeated behavioural surveillance in 2013–17. Lancet HIV 2018; 5:e448–56. 29885813 10.1016/S2352-3018(18)30072-9

[9] Chow EPF, Grulich AE, Fairley CK. Epidemiology and prevention of sexually transmitted infections in men who have sex with men at risk of HIV. Lancet HIV 2019; 6:e396–405. 31006612 10.1016/S2352-3018(19)30043-8

[10] Peeling RW, Mabey D, Kamb ML, Chen XS, Radolf JD, Benzaken AS. Syphilis. Nat Rev Dis Primers 2017; 3:17073. 29022569 10.1038/nrdp.2017.73 PMC5809176

[11] Kent ME, Romanelli F. Reexamining syphilis: an update on epidemiology, clinical manifestations, and management. Annals of Pharmacother 2008; 42:226–36. 10.1345/aph.1K086 18212261

[12] Hook EW 3rd . Syphilis. The Lancet 2017; 389:1550–7. 10.1016/S0140-6736(16)32411-4 27993382

[13] Stoltey JE, Cohen SE. Syphilis transmission: a review of the current evidence. Sex Health 2015; 12:103–9. 25702043 10.1071/SH14174 PMC5973824

[14] Cornelisse VJ, Chow EPF, Latimer RL, et al Getting to the bottom of it: sexual positioning and stage of syphilis at diagnosis, and implications for syphilis screening. Clin Infect Dis 2020; 71:318–22. 31420649 10.1093/cid/ciz802

[15] Nyitray AG, Hicks JT, Hwang LY, et al A phase II clinical study to assess the feasibility of self and partner anal examinations to detect anal canal abnormalities including anal cancer. Sex Transm Infect 2018; 94:124–30. 28835533 10.1136/sextrans-2017-053283 PMC6173609

[16] Fairley CK, Chow EPF, Simms I, Hocking JS, Ong JJ. Accessible health care is critical to the effective control of sexually transmitted infections. Sex Health 2022; 19:255–64. 35760765 10.1071/SH22042

[17] Aung ET, Fairley CK, Ong JJ, et al Adherence to weekly anal self-examination among men who have sex with men for detection of anal syphilis. Front Med (Lausanne) 2022; 9:941041. 35979212 10.3389/fmed.2022.941041 PMC9376231

[18] Hillman RJ, Berry-Lawhorn JM, Ong JJ, et al International anal neoplasia society guidelines for the practice of digital anal rectal examination. J Low Genit Tract Dis 2019; 23:138–46. 30907777 10.1097/LGT.0000000000000458

[19] Aung ET, Chow EPF, Fairley CK, et al Preferences of men who have sex with men for performing anal self-examination for the detection of anal syphilis in Australia: a discrete choice experiment. Lancet Reg Health West Pac 2022; 21:100401. 35243457 10.1016/j.lanwpc.2022.100401 PMC8873922

[20] Aung ET, Fairley CK, Ong JJ, et al Exploring the attitudes of men who have sex with men on anal self-examination for early detection of primary anorectal syphilis: a qualitative study. BMC Infect Dis 2021; 21:982. 34544383 10.1186/s12879-021-06686-4 PMC8453991

[21] Aung ET, Fairley CK, Ong JJ, et al A cross-sectional survey on attitudes of men who have sex with men towards anal self-examination for detection of anal syphilis. Sci Rep 2022; 12:8962. 35624185 10.1038/s41598-022-12881-3 PMC9142515

[22] Nyitray AG, D'Souza G, Stier EA, Clifford G, Chiao EY. The utility of digital anal rectal examinations in a public health screening program for anal cancer. J Low Genit Tract Dis 2020; 24:192–6. 31972661 10.1097/LGT.0000000000000508 PMC7147422

[23] Ong JJ, Bourne C, Dean JA, et al Australian sexually transmitted infection (STI) management guidelines for use in primary care 2022 update. Sex Health 2023; 20:1–8. 36356948 10.1071/SH22134

[24] The Australian Centre for Disease Control . Syphilis (less than 2 years duration) – Surveillance case definition. https://www.cdc.gov.au/resources/publications/surveillance-case-definition-syphilis-under-2-years. Accessed 17 December 2025.

[25] Kellogg TA, Loeb L, Dilley J, Adler B, Louie BT, McFarland W. Comparison of three methods to measure HIV incidence among persons seeking voluntary, anonymous counseling and testing. J Acquir Immune Defic Syndr 2005; 39:112–20. 15851921 10.1097/01.qai.0000144444.44518.a3

[26] Magnuson HJ, Thomas EW, Olansky S, et al Inoculation syphilis in human volunteers. Medicine (Baltimore) 1956; 35:33–82. 13296652 10.1097/00005792-195602000-00002

[27] Forrestel AK, Kovarik CL, Katz KA. Sexually acquired syphilis: historical aspects, microbiology, epidemiology, and clinical manifestations. J Am Acad Dermatol 2020; 82:1–14. 30986477 10.1016/j.jaad.2019.02.073

[28] Ong JJ, Towns JM, Chen MY, Fairley CK. Occult syphilitic chancres in the rectum and oropharynx. Aust Fam Physician 2017; 46:673–5. 28892599

[29] Shu Z, Zhao J-l, Deng X-f, Ge C-j, Sun F, Zee C-S. Primary chancre in the rectum: a report of rare case of syphilis. Radiol Infect Dis 2014; 1:29–31.

[30] Butovskaya M, Burkova V, Apalkova Y, et al Sex, population origin, age and average digit length as predictors of digit ratio in three large world populations. Sci Rep 2021; 11:8157. 33854119 10.1038/s41598-021-87394-6 PMC8046776

[31] Singh AE, Romanowski B. Syphilis: review with emphasis on clinical, epidemiologic, and some biologic features. Clin Microbiol Rev 1999; 12:187–209. 10194456 10.1128/cmr.12.2.187 PMC88914

[32] Chow EPF, Hocking JS, Ong JJ, et al Brief report: changes in PrEP use, sexual practice, and use of face mask during sex among MSM during the second wave of COVID-19 in Melbourne, Australia. J Acquir Immune Defic Syndr 2021; 86:153–6. 33433122 10.1097/QAI.0000000000002575 PMC7808277

[33] Chow EPF, Hocking JS, Ong JJ, Phillips TR, Fairley CK. Sexually transmitted infection diagnoses and access to a sexual health service before and after the national lockdown for COVID-19 in Melbourne, Australia. Open Forum Infect Dis 2021; 8:ofaa536–NaN. 33506064 10.1093/ofid/ofaa536 PMC7665697

