# Supplementary material for: Digital Anorectal Examination to Self-detect Primary Syphilis: A Prospective Cohort Study
Source: J Infect Dis. 2025 Dec 11;233(3):e696–705. doi: 10.1093/infdis/jiaf628 (PMC13017385; doi:10.1093/infdis/jiaf628)
Supplement: jiaf628_Supplementary_Data [file jiaf628_supplementary_data.zip › TableS1.docx]

**Table S1.** Performing DARE among 125 men at week 48

| **Details** | n | % |  |
| --- | --- | --- | --- |
| Positions used when performing DARE throughout the study: |  |  |  |
| *Squatting* | 56 | 44.8 |  |
| *Standing on a stool or toilet* | 41 | 32.8 |  |
| *Lying on the side* | 28 | 22.4 |  |
| Items used when performing DARE throughout the study ***^†^***: |  |  |  |
| *Mirror* | 86 | 68.8 |  |
| *Lubricant* | 84 | 64.8 |  |
| *Water* | 60 | 48.0 |  |
| *Soap* | 40 | 32.0 |  |
| *Gloves* | 15 | 12.0 |  |
| Location where DARE was performed throughout the study ***^†^***: |  |  |  |
| *Showers* | 79 | 63.2 |  |
| *Bathroom/toilet* | 58 | 46.4 |  |
| *Bedroom* | 31 | 24.8 |  |
| Reasons for not performing DARE***^†^***: |  |  |  |
| *I* *was consistent with DARE* | 74 | 59.2 |  |
| *I had forgotten to do it* | 40 | 32.0 |  |
| *I was busy with other commitments* | | 31 | 24.8 |
| *I did not have any symptoms* | 29 | 23.2 |  |
| *Did not have receptive anal sex* | 20 | 16.0 |  |
| *Did not have other anorectal exposures, e.g., being rimmed* | 11 | 8.8 |  |
| *Performing DARE was uncomfortable* | 7 | 5.6 |  |
| *I was concerned about finding abnormalities* | 2 | 1.6 |  |
|  |  |  |  |
| Performed DARE on their partner(s) | 44 | 35.2 |  |
| Number of partners on whom DARE was performed in the past 3 months, median (IQR) | 2 (1-4) |  |  |
| Number of men who found abnormalities after performing DARE on partner(s) | 4 | 9.1 |  |
| Abnormalities found: |  |  |  |
| *Ulcer(s)* | 1 | 25.0 |  |
| *Pain* | 1 | 25.0 |  |
| *Other:* |  |  |  |
| *Patch of skin* | 1 | 25.0 |  |
| *Haemorrhoid* | 1 | 25.0 |  |
| Men who talked to a partner after they noticed abnormalities on their partner | 3 | 75.0 |  |
| Partner's actions after talking about the abnormalities: |  |  |  |
| *Consulted a doctor or a nurse at MSHC* | 1 | 33.3 |  |
| *Did not do anything* | 1 | 33.3 |  |
| *Other:* |  |  |  |
| *Partner confirmed it was a haemorrhoid* | 1 | 33.3 |  |

IQR, interquartile range

DARE, digital anorectal examination

***^†^***Multiple answers were permitted, and proportions may exceed 100%
