# Supplementary material for: Digital Anorectal Examination to Self-detect Primary Syphilis: A Prospective Cohort Study
Source: J Infect Dis. 2025 Dec 11;233(3):e696–705. doi: 10.1093/infdis/jiaf628 (PMC13017385; doi:10.1093/infdis/jiaf628)
Supplement: jiaf628_Supplementary_Data [file jiaf628_supplementary_data.zip › TableS2_20251204.docx]

**Table S2.** Preferences and suggestions regarding DARE among 125 men at Week 48

| **Details** | n | % |
| --- | --- | --- |
| **Preferences** |  |  |
| What is the reasonable number of times DARE should be performed per year? Median (IQR) | 40 (25-50) |  |
| Would you continue to perform DARE if it is recommended for detecting syphilis?* |  |  |
| *Yes* | 97 | 77.6 |
| *No* | 3 | 2.4 |
| *Unsure* | 25 | 20.0 |
| Reasons for continuing DARE** |  |  |
| *Health reasons (detect conditions early, seek treatment, maintain sexual health)* | 80 | 88.9 |
| *Easy and convenient method* | 16 | 17.8 |
| **Suggestions** |  |  |
| What are some other that can help men perform DARE besides an instructional video? ***^†^*** |  |  |
| *A website with pictorial instructions* | 66 | 52.8 |
| *Online videos from a trusted source* | 57 | 45.6 |
| *Pictorial instructional poster* | 57 | 45.6 |
| *Instructions from a doctor* | 52 | 41.6 |
| *Videos of a doctor or a nurse using a training model, or a live person* | 46 | 36.8 |
| *Instructions from a doctor using a training model in person* | 34 | 27.2 |
| *Instructions from a doctor and followed by DARE by a doctor* | 29 | 23.2 |
| *Learning from partners or friends* | 26 | 20.8 |
| *People will not need resources for this* | 6 | 4.8 |
| *Other:* |  |  |
| *Smartphone reminder app from trusted source* | 1 | 0.8 |
| What information would be useful for other men to learn about DARE?***^†^*** |  |  |
| *Information on what a syphilis lesion looks like* | 84 | 68.0 |
| *Information on what a syphilis lesion feels like* | 78 | 62.4 |
| *Information on what a healthy anus should look like* | 74 | 59.2 |
| *Information on how to perform DARE* | 72 | 57.6 |
| *STI symptoms on the anus, such as warts and herpes* | 66 | 52.8 |
| *Information about non-STI symptoms of anus (e.g., haemorrhoids)* | 65 | 52.0 |
| *Information on the importance of DARE* | 60 | 48.0 |
| *Information about symptoms of anal cancer* | 52 | 41.6 |
| *Information on where to do a self-examination* | 38 | 30.4 |
| *All of the above* | 63 | 50.4 |

DARE, digital anorectal examination

IQR, interquartile range

STI, sexually transmissible infection

*Three men did not provide reasons as to why they would not continue with DARE

**Seven men did not provide reasons as to why they would continue with DARE

***^†^***Multiple responses were permitted, and proportions may exceed 100%

Note: Participants’ reasons to continue with DARE were provided in response to an open-ended question
