# Supplementary figures and images for: Digital Anorectal Examination to Self-detect Primary Syphilis: A Prospective Cohort Study
Source: J Infect Dis. 2025 Dec 11;233(3):e696–705. doi: 10.1093/infdis/jiaf628 (PMC13017385; doi:10.1093/infdis/jiaf628)

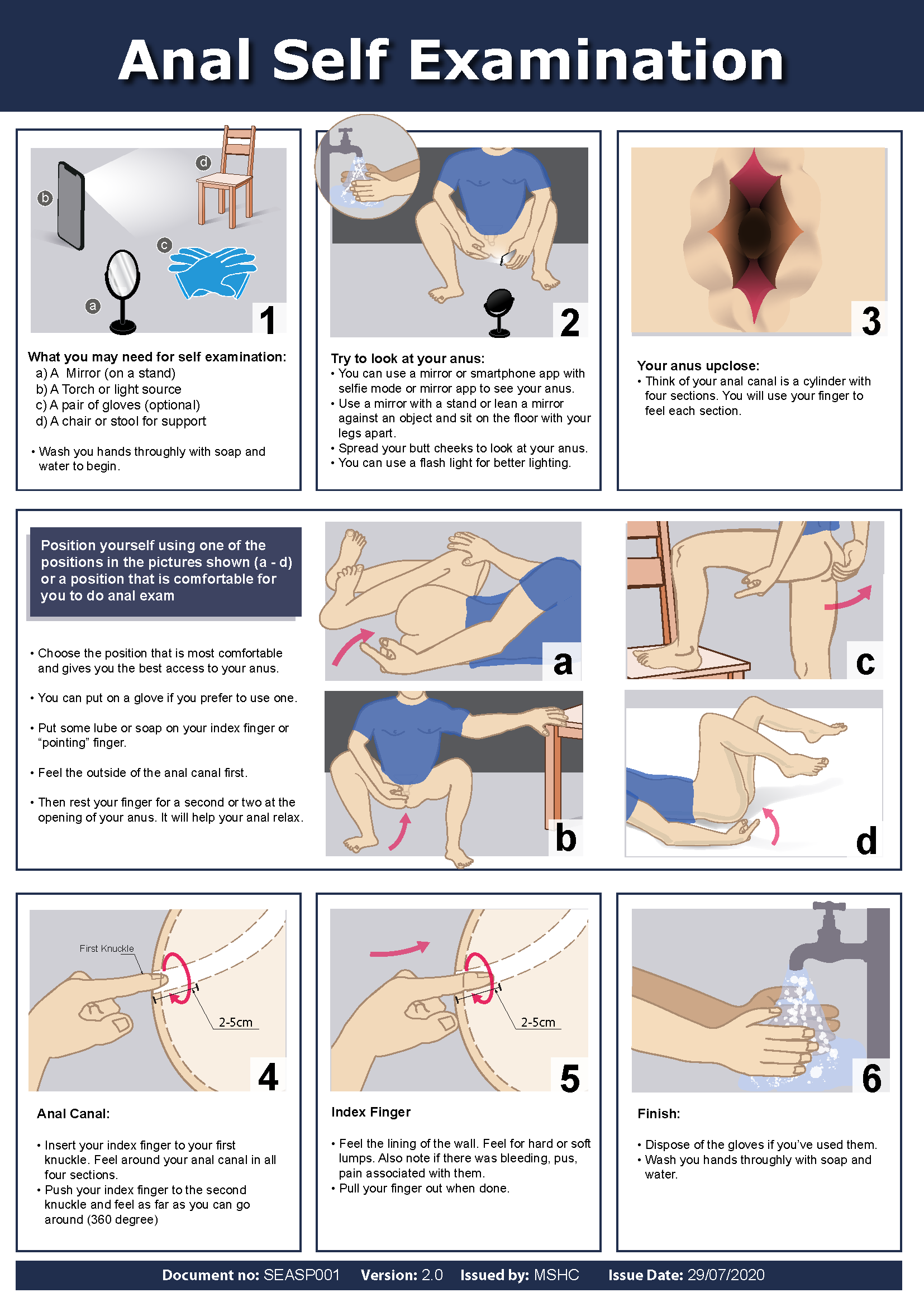


**Figure S1**. Instructions for digital anorectal examination

Supplement: jiaf628_Supplementary_Data [file jiaf628_supplementary_data.zip › SupplementaryFigureS1.docx]
